# Exploring the Dynamical Nature of Intermolecular Hydrogen Bonds in Benzamide, Quinoline and Benzoic Acid Derivatives

**DOI:** 10.3390/molecules27248847

**Published:** 2022-12-13

**Authors:** Kamil Wojtkowiak, Aneta Jezierska

**Affiliations:** Faculty of Chemistry, University of Wrocław, ul. F. Joliot-Curie 14, 50-383 Wrocław, Poland

**Keywords:** hydrogen bond, non-covalent interactions, spectroscopic signatures, CPMD, PIMD, QTAIM, SAPT

## Abstract

The hydrogen bonds properties of 2,6-difluorobenzamide, 5-hydroxyquinoline and 4-hydroxybenzoic acid were investigated by Car–Parrinello and path integral molecular dynamics (CPMD and PIMD), respectively. The computations were carried out in vacuo and in the crystalline phase. The studied complexes possess diverse networks of intermolecular hydrogen bonds (N-H…O, O-H…N and O-H…O). The time evolution of hydrogen bridges gave a deeper insight into bonds dynamics, showing that bridged protons are mostly localized on the donor side; however, the proton transfer phenomenon was registered as well. The vibrational features associated with O-H and N-H stretching were analyzed on the basis of the Fourier transform of the atomic velocity autocorrelation function. The spectroscopic effects of hydrogen bond formation were studied. The PIMD revealed quantum effects influencing the hydrogen bridges providing more accurate free energy sampling. It was found that the N…O or O…O interatomic distances decreased (reducing the length of the hydrogen bridge), while the O-H or N-H covalent bond was elongated, which led to the increase in the proton sharing. Furthermore, Quantum Theory of Atoms in Molecules (QTAIM) was used to give insight into electronic structure parameters. Finally, Symmetry-Adapted Perturbation Theory (SAPT) was employed to estimate the energy contributions to the interaction energy of the selected dimers.

## 1. Introduction

Hydrogen bond (HB) can be considered to be the most important type of non-covalent interaction [1]. It is important to emphasize that hydrogen bonds are of great importance for the properties of water, the binding of drugs to receptors or the stability of macromolecules [2,3]. Furthermore, HBs are some of the most important factors that affect the packing in crystals [4]. A hydrogen bond is usually defined as X-H…:Y, the interaction of a bridging hydrogen attached to an electronegative donor atom (denoted as X) with another electron-rich species (denoted as Y) [5]. A common feature of most types of hydrogen bonds is the elongation of the X-H covalent bond with the co-existing redshift in the X-H stretching vibrations and a decrease in H…Y distance. However, this classical definition does not encompass the whole diversity of hydrogen bonds. As shown, HBs can also be formed with carbon as a hydrogen donor or acceptor [1]. Theoretical studies of C-H proton donors with benzene and ethylene oxide species as acceptors have shown that redshift is not a definitive characteristic of all HBs—in the cited studies, the authors have demonstrated the existence of so-called anti-hydrogen or blue-shifting hydrogen bonds, in which hydrogen bond formation is accompanied by the C-H covalent bond contraction and an increase in its stretch (blue shift) [6,7]. A closely related group of non-covalent interactions, which are based on the same physical principles, is the so-called σ-hole bond family [8,9,10,11,12,13]. The σ-hole concept is related to the depletion of electron density on the bridge atom at the extension of its covalent bond, X-A (A denotes the bridge atom and X is any electronegative species).

Due to a local increase in molecular electrostatic potential (MEP), an atom can participate in a highly directional interaction with Lewis bases [14,15]. These electrostatically driven interactions are named after the family from which the bridge atom is derived [16]. Among the most well known are halogen, chalcogen and pnicogen bonds [17,18,19,20,21,22,23]. In general, the strength of these interactions increases with the increasing electronegativity, polarizability and basicity of the donor atom, bridge atom and Lewis base, respectively [24,25]. Redshift is usually a reliable measure of their strength, however, the change in their X-A stretching frequency is less correlated with the interaction strength compared to HBs [18,26]. Moreover, the heavier and more polarizable the bridge atom is, the smaller the co-occurring redshift [25].

Theoretical methods such as the quantum theory of atoms in molecules (QTAIM) [27], Reduced Density Gradient (RDG) [28], Electron Localization Function (ELF) [29] or various perturbational or variational energy-decomposition schemes [30,31] have proven to be invaluable in the detailed characterization of HBs and other secondary bonds [24,32]. From a theoretical perspective, there is also a way to characterize hydrogen bonding and it is usually performed using the QTAIM conceptual apparatus—the indicators of the presence of the HB in the examined system are: (i) the bond path between the proton donor and acceptor atoms with the Bond Critical Point (BCP) located on it; and (ii) relatively small electron density at the BCP between atoms (about an order to even two orders smaller than $\rho_{BCP}$ for typical covalent bonds) with the Laplacian values close to zero. Considering the energy-decomposition of the hydrogen bonds it is usually described as mainly covalent in nature, especially in the case of strong HBs with an interaction energy above 24 kcal * mol^−1^ [33,34]. In this context, the term “covalent” is associated with a shortening of the H…Y distance and an increase in the importance of the induction and dispersion terms (in terms of the nomenclature used in the SAPT energy-partitioning scheme [30]). On the contrary, weak hydrogen bonds are described as interactions in which the electrostatics play the most important role [35].

However, it should not be forgotten that biological or chemical systems are inherently dynamic, hence the above-mentioned, so-called static approaches which, despite being very useful, can only provide information about one particular arrangement of atoms in the complex. Thus, Car–Parrinello molecular dynamics (CPMD) [36] and path integral molecular dynamics (PIMD) [37] are among the most often employed dynamical approaches to study H/D isotope effects [38], non-covalent interactions and hydrogen bridges in particular [39,40,41]. Furthermore, the quantization of nuclei using PIMD allows one to take into account the quantum nature of the examined system—the hydrogen atom is particularly sensitive to quantum effects, even at a standard temperature due to its small mass, and thus has a relatively large value of thermal de Broglie wavelength compared to other atoms. Noteworthy is the fact that the quantum-tunneling phenomena (which can be efficiently studied using path integration techniques [42,43]) are important in biochemistry and are associated with enzyme-mediated electron or hydrogen transfer [44,45]. Nowadays, attempts are being made to study the quantum effects at the ligand–receptor binding site. An example of this can be found in the work where binding affinities for histamine receptor ligands were studied [46]. Another important aspect is associated with the spectroscopic features’ investigation on the basis of the CPMD method. The vibrational properties could be studied using standard approaches, that is, the Fourier transformation of the autocorrelation function of atomic velocity or dipole moment. However, it is also possible to apply a method, which enabled the a posteriori inclusion of quantum effects to the O-H, N-H stretching etc. The method was successfully used in studies where strongly anharmonic systems were investigated [47,48,49,50].

Due to the aforementioned reasons, we decided to take a hybrid approach in order to characterize in detail the HBs present in the studied compounds—the dynamical features as well as detailed static characteristics were taken into account. Interactions for dimers extracted from the crystal were quantified and assessed using the SAPT and QTAIM approaches. The metric parameters and spectroscopic signatures of the investigated compounds were obtained and thoroughly analyzed. Obtaining quantum statistics for the nuclear degrees of freedom via PIMD application allowed to estimate the importance of quantum effects in the description of intermolecular HBs, when compared to the classical-quantum CPMD approach.

In the current study, we investigated three aromatic compounds from the benzamide, quinoline and benzoic acid groups. The choice was dictated by the network of hydrogen bonds present in the crystal structures [51]. Benzamide is a derivative of benzoic acid. Some substituted benzamides are well-known commercial drugs, e.g., procainamide, imatinib and veralipride [52,53,54]. Benzamides are still an attractive group of compounds, especially in drug design, where very often their derivatives are taken into consideration as compounds that show a specific type of biological activity. Therefore, they are studied both experimentally and by molecular modeling methods, e.g., [55,56,57,58,59]. We chose 2,6-difluorobenzamide, which is a metabolite of pesticide diflubenzuron [60], to theoretically study its hydrogen bonding network. It was found that benzamide as well as 2,6-difluorobenzamide can form mutual intermolecular hydrogen bonds. However, the extended amide…amide dimer synthon in benzamide can form a network of HBs via NH_2_ group. Concerning the 2,6-difluorobenzamide, the presence of fluorine atoms allows the formation of other intermolecular interactions [51]. The next compound chosen for our theoretical investigations is 5-hydroxyquinoline, where the O-H…N intermolecular hydrogen bond is present as the strongest intermolecular interaction, however, C-H…O interaction was noted in the crystalline phase [35]. Taking into account the fact that quinoline and its derivatives have diverse applications, e.g., in medicine as drugs, compounds exhibiting various biological activity, as dyes, and as solvents [61,62,63,64,65,66,67], it is of interest to investigate the properties of hydrogen bonds in the class of compounds. The last compound taken into account was 4-hydroxybenzoic acid, which is the simplest aromatic carboxylic acid. Benzoic acid occurs naturally in many plants [68]. Its salts are used as food and cosmetics preservatives [69]. Generally speaking, it is an important precursor for the industrial synthesis of many other organic substances [70,71]. 4-hydroxybenzoic acid is primarily known as the basis for the preparation of its esters (parabens), which are used as preservatives in, e.g., cosmetics [72,73]. In our case, it was interesting to explore the dynamical nature of intermolecular hydrogen bonds, wherein the carboxylic as well as hydroxy groups were involved [51].

We hope that our research contributes to the knowledge of hydrogen bond dynamics, and will help with the rational design of new derivatives with specific properties. Therefore, the main objective of this research is to perform multi-factor studies of non-covalent interactions in the examined compounds. In order to reproduce the dynamical nature of a hydrogen bonds network, we employed the Car–Parrinello molecular dynamics [36]. To be able to make comparisons the time evolution simulations were performed in vacuo and in the crystalline phase. The nuclear quantum effects (NQEs) was taken into account and path integral molecular dynamics (PIMD) simulations were performed for this purpose [37,74]. The quantum theory of atoms in molecules (QTAIM) [27] was applied for electron density topological studies enabling the estimation of the interaction strength and the detection of weaker interactions. An application of symmetry-adapted perturbation theory (SAPT) [30] method allowed the energy decomposition in the studied dimers.

## 2. Computational Methodology

### 2.1. Car–Parrinello Molecular Dynamics (CPMD)

**Figure 1:**
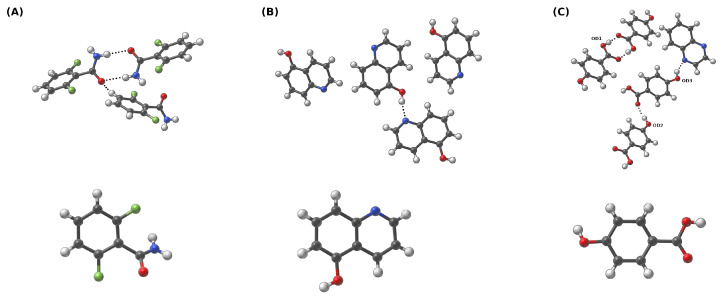
Crystalline (first row) and gaseous phases (second row) of (**A**) 2,6-difluorobenzamide, (**B**) 5-hydroxyquinoline, and (**C**) co-crystal of 4-hydroxybenzoic acid and quinoxaline. For clarity, the oxygen donor atoms for (**C**) are denoted as OD1, OD2 and OD3—this adopted nomenclature is used throughout the study. Dotted line indicates the intermolecular hydrogen bond. Color coding: white—hydrogen, grey—carbon, red—oxygen, blue—nitrogen, green—fluorine.

**Figure 2:**
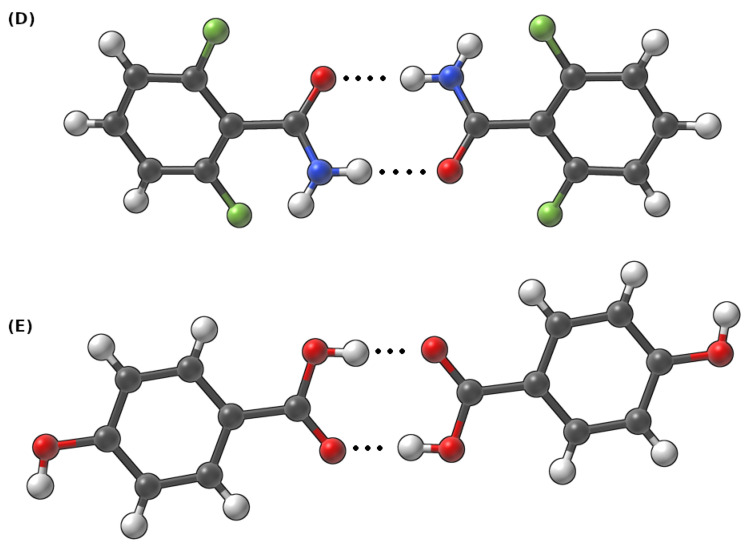
Dimers taken from the crystalline phase of (**D**) 2,6-difluorobenzamide and (**E**) 4-hydroxybenzoic acid to study intermolecular hydrogen bonds. Dotted line indicates the intermolecular hydrogen bond.

The CPMD [36] computations were performed in the crystalline phase for three crystals taken from the Cambridge Crystallographic Data Centre (CCDC) [75]. Their CCDC codes are as follows: (A)—919101, (B)—908102 and (C)—908103 [51]. The molecular structures of the studied complexes are presented in Figure 1 and Figure 2. Two different sets of structures were chosen to thoroughly describe the intermolecular hydrogen bonds present in these systems. The simulations of the monomers of the (A), (B) and (C) were conducted in the gas phase with the box edges set to: a = b = c = 15 Å. The Perdew–Burke–Ernzerhof functional (PBE) [76] and the norm-conserving Troullier-Martins pseudopotentials [77] were applied. The plane-wave kinetic energy cutoff was set to 100 Ry. The models for crystalline-phase simulations were constructed based on experimental data—the details of which are presented in Table 1. The periodic boundary conditions (PBCs) with real-space electrostatic summations for the eight nearest neighbors in each direction were employed during the crystalline phase computations. Additionally, simulations were performed for dimers in the gas phase as well. The sizes of the corresponding simulation boxes were benchmarked and adjusted to calculate the Hartree potential using the Hockney solver of the Poisson equation. The edges of the corresponding boxes for the dimers were equal to: a = 18 Å b = 21 Å c = 18 Å for the (D) dimer and a = 22 Å b = 18 Å c = 18 Å for the (E) dimer.

Subsequently, Car–Parrinello molecular dynamics (CPMD) [36] simulations were performed with the CPMD 4.3 suite of programs [78]. During the computations, the time step was set to 3 a.u., while the fictitious electron mass (EMASS) parameter was equal to 400 a.u. in both phases. The temperature was controlled by the Nosé–Hoover thermostat [79,80] and it was set to 297 K. The obtained CPMD trajectories were divided into equilibration (first 10,000 steps were excluded from the data analysis) and the production run. The CPMD production runs of the crystalline models were collected for ca. 72 ps and 42 ps for crystalline and gas-phase systems, respectively (monomers dynamics in the gas phase was simulated only to obtain the spectroscopic signatures of the O-H, C-H and N-H functional groups). Molecular dynamics corresponding to the models chosen to study dimer interactions were propagated for ca. 45 ps. The post-processing included the analysis of metric and spectroscopic properties. The vibrational features were studied using the Fourier transform of the atomic velocity autocorrelation function. This type of analysis enabled the decomposition of the computed IR spectra. The O-H and N-H stretching was obtained to give a deeper insight into spectroscopic signatures in the investigated hydrogen bridges. The metric parameters analysis was performed with the assistance of the VMD 1.9.3 program [81], while the Fourier transform power spectra of the atomic velocity were computed using home-made scripts. The graphs were obtained with the Gnuplot [82] program. The experimental unit cells were visualized and analyzed using the Mercury [83] program. The visualizations presented in Figure 1 and Figure 2 were prepared in the SAMSON suite of programs [84].

### 2.2. Path Integral Molecular Dynamics (PIMD)

The quantum nature of the nuclear motion in the crystals was investigated using the path integral molecular dynamics (PIMD) approach [37,74]. The simulations were carried out in the gas and crystalline phases using the models prepared for the CPMD runs. The electronic structure setup was the same as described in the previous subsection. The computations were performed at 297 K temperature controlled by Nosé–Hoover thermostat [79]. The staging representation of the path integral propagator was used [37] and eight Trotter replicas (P = 8) were applied for imaginary time path integration. The initial 5000 steps of the simulation time were excluded from the analysis and treated as an equilibration phase. The trajectories of length 21 and 11 ps relative to the first and the second group of the structures under study, respectively, were collected and taken as production runs (11 ps in the case of (C) in the solid state). The data analysis was carried out with the assistance of home-made scripts. The PIMD simulations were performed with the CPMD version 4.3 program [78]. The Gnuplot program [82] was applied for the histogram preparation.

### 2.3. Quantum Theory of Atoms in Molecules (QTAIM)

Quantum theory of atoms in molecules (QTAIM) was applied to X-ray structures as well as to structures optimized at the ωB97XD/def2-TZVP level of theory [85,86] using the Gaussian 16 Rev. C.0.1 suite of programs [87]. The wavefunctions for further electronic structure analysis were obtained with the same computational setup. This part of the investigations was only performed for the gas phase models. The QTAIM analysis was performed with the assistance of the MultiWFN 3.8 program [88,89]. The graphical presentation of the obtained results was prepared using the VMD 1.9.3 program [81].

### 2.4. Symmetry-Adapted Perturbation Theory (SAPT)

Symmetry-adapted perturbation theory (SAPT) [30] was applied to gain an insight into the interaction energy between two studied molecules forming a dimer. The approximation of four-index integrals was carried out using the density-fitting technique (RI and JKI) with aug-cc-pVDZ (aDZ) [90] as auxiliary basis sets. The examined dimers were separated into two monomers in order to fulfill the conditions needed to eliminate the basis set superposition error (BSSE) [91]. The interaction energies were obtained at the SAPT2+/aDZ [92] level of theory. All calculations were performed using the Psi4 1.3.2 suite of programs [93].

## 3. Results and Discussion

### 3.1. Spectroscopic and Metric Parameters Associated with Intermolecular Hydrogen Bonds: Gas Phase vs. Crystalline Phase in the Light of Car–Parrinello Molecular Dynamics (CPMD) and Path Integral Molecular Dynamics (PIMD)

**Figure 3:**
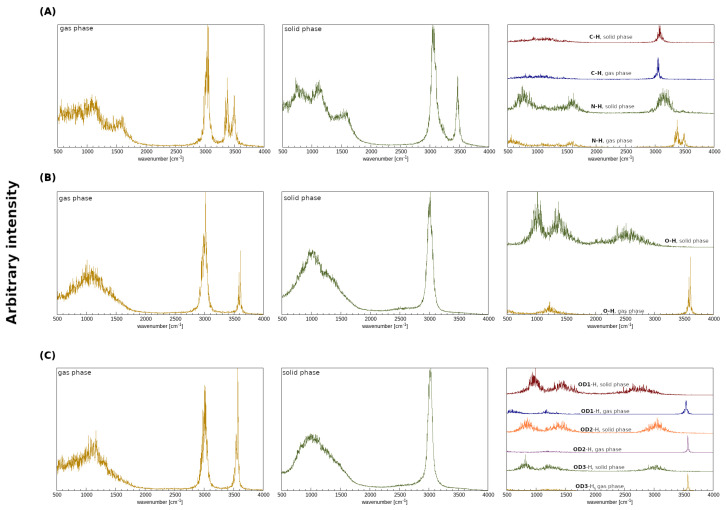
Atomic velocity power spectra obtained from the CPMD simulations. Left panel: the whole atomic spectra in the gas phase. Middle panel: the whole atomic spectra in the crystalline phase. Right panel: the contribution of the bridged protons in the crystalline and in the gaseous phases. (**A**) 2,6-difluorobenzamide, (**B**) 5-hydroxyquinoline and (**C**) 4-hydroxybenzoic acid.

The gas phase as well as the crystalline phase simulations were carried out for 2,6-difluorobenzamide, 5-hydroxyquinoline and 4-hydroxybenzoic acid to investigate the intermolecular hydrogen bonds. Car–Parrinello molecular dynamics and Path Integral molecular dynamics allowed for the quantitative and qualitative description of the spectroscopic and geometric features of the studied compounds. In Figure 1, the models used for the crystalline phase (based on crystal structures (A) 919101, (B) 908102 and (C) 908103 [51]) and gas phase molecular dynamics simulations are presented. In order to obtain a full spectroscopic description of the aforementioned models, all simulations were carried out in the gas and in the crystalline phases. It is worth emphasizing that the results obtained in vacuo served as a reference for the O-H and N-H stretching discussion. On the basis of the Fourier transform of the atomic velocity autocorrelation function, the classical vibrational spectrum was obtained (see Figure 3). A major advantage of the methodology used is the ability to estimate the individual contributions of selected atoms to the entire spectrum—these are shown in the third column of the Figure 3. Due to the characteristics of the CPMD method (nuclei dynamics is inherently classical), the Fermi resonance, and thus the splitting of the bands of nearly identical energies and symmetries as well as tunneling phenomena, cannot be observed. Moreover, the classical amplitudes of motion at 297 K allow for sampling a narrower part of the potential energy surface than the true quantum particle with its nuclear wavefunction delocalization. This leads in many cases to the underestimation of the anharmonicity and is another factor of deviation between the CPMD-derived X-H stretching position and the experimental spectrum. It is therefore a good idea to concentrate not on the absolute wavenumbers, but on the shifts between the hydrogen-bonded protons and free protons. In the case of the results shown in Figure 3 (see the first and the second panels), the presence of the two regions characterized by the increased intensities can be seen: the region of the deformation vibrations, from ca. 500 to 1800 cm^−1^ in the solid and in the gas phase, as well as the region of stretching vibrations that extend from 2800 to 3700 cm^−1^. The former can be attributed to the heavy atom oscillations, whereas the latter is the signature region for the protons, including those involved in the hydrogen bond formation. The third panel corresponds to the particular protons involved in the HBs in the crystalline phase. In addition, the gas phase results presented in the third panel show the characteristic, sharp stretching modes of O-H, N-H and C-H not involved in the hydrogen bonding. For (A), one may observe the blueshift of the C-H stretching vibration (from ca. 3050 to 3150 cm^−1^) and thus CD-H…OA can be regarded as an anti-hydrogen bond [6,94]. In the case of ND-H…OA, it is visible that the N-H band for the solid phase is shifted towards lower wavenumber values (redshift). This is evidence for the ND-H weakening and the accompanying contraction and strengthening of the ND-H…OA bond, which can be interpreted as the charge transfer from the proton acceptor to the antibonding orbital of the ND-H and as a sign of the hydrogen bond formation. In the case of (B) and (C), similar observations can be made—the O-H stretching is redshifted in the solid phase, when compared to the gas phase. In summary, the spectroscopic features obtained allow us to conclude that the hydrogen bonds were formed in each of the compounds studied in the solid state.

**Figure 4:**
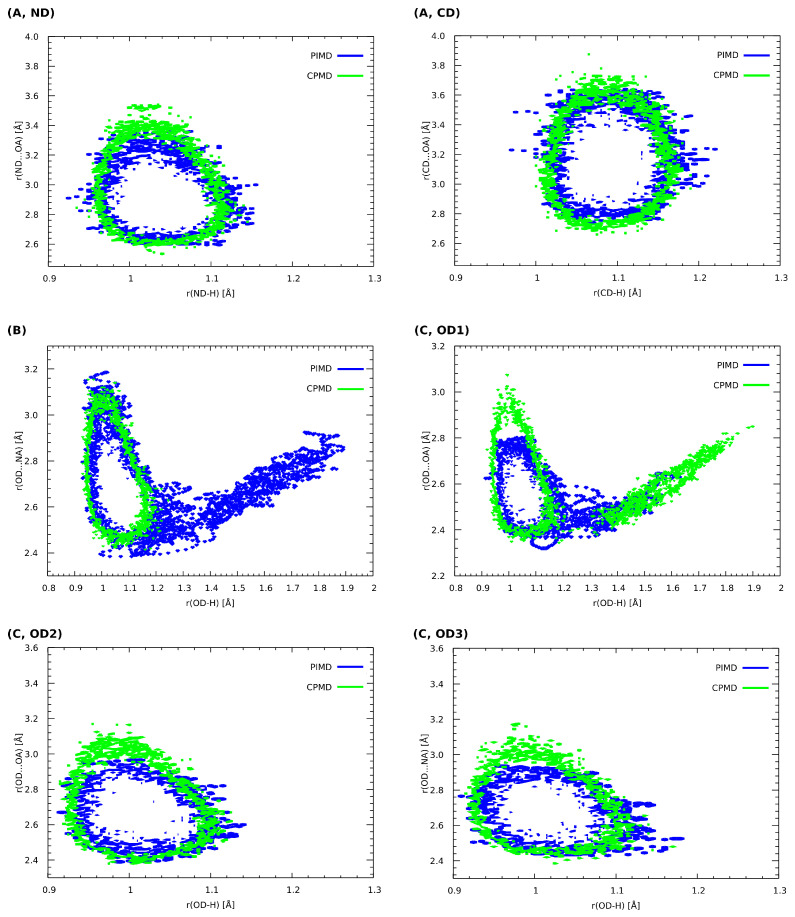
Histograms presenting the relationship between the length of the corresponding donor–proton covalent bonds as well as the distance between the proton and its acceptor. CPMD vs. PIMD in the crystalline phase. (**A**) 2,6-difluorobenzamide, (**B**) 5-hydroxyquinoline and (**C**) 4-hydroxybenzoic acid. ND, OD and CD indicate proton donors. Probability density isocontours drawn at the 1 Å${}^{-2}$ value.

The analysis of the Car–Parrinello (CPMD) and path integral molecular dynamics (PIMD) trajectories enables one to obtain a probability distribution of bridge proton positions in the studied compounds (see Figure 4). The characteristics of proton motion in all of hydrogen bridges highlighted in Figure 1 were obtained. Considering the impact of the NQEs on the proton behavior, one can observe that, in comparison with CPMD, the distance between the proton donor and its acceptor is insignificantly shortened for (A) (decrease of ca. 0.1 Å in each case). The same observation can be made for the ND-H and CD-H values—in these cases, the quantization of the nuclei does not change the proton dynamics. Indeed, both aforementioned hydrogen bonds cannot be regarded as strong hydrogen bonds—in the first case (A, ND), the proton donor is an amide group, with a behavior strongly altered by the presence of two fluorine substituents in -ortho positions. In the second case, the proton donor is the aromatic carbon atom and thus the HB can be considered weak (this is an example of the blue-shifting hydrogen bond, as we argued in the previous section). Different dynamical characteristics of the bridged proton were noticed for 5-hydroxyquinoline as well as the OD1 of the 4-hydroxybenzoic acid and quinoxaline co-crystal. For (B), one can observe that the proton mobility increased significantly, when the dynamics was performed in the PIMD scheme. On the contrary, the NQEs for the (C, OD1) provided further stabilization of the hydrogen atom at the donor side; in this case, the CPMD simulations indicated that the proton is more delocalized. Here, proton transfer, from the donor to the acceptor site in the hydrogen bond, has occurred, which can be easily discerned by the presence of the distinctive “tail” that extends towards larger r(OD-H) values. In the case of OD2-H…OA hydrogen bond (C, OD2), the only difference concerns the negligible shortening of OD2…OA distance in PIMD, when compared to the CPMD. For (C, OD3), one may observe that the NQEs induce minimal r(OD3-H) covalent bond elongation and the accompanying contraction of the r(OD3…NA) distance. Summarizing, it can be noted that the inclusion of the NQEs for the presented set of compounds results in changes in the quantitative as well as qualitative nature. The latter are especially pronounced for (B), where the free energy surface was sampled by the proton more efficiently and for (C, OD1) case, where the NQEs inclusion resulted in a more localized behaviour of the proton in the hydrogen bridge. In all studied cases, with the exception of the blue-shifting (A, CD) bond, a decrease in the donor–acceptor distance is accompanied by increased proton delocalization and sharing.

**Figure 5:**
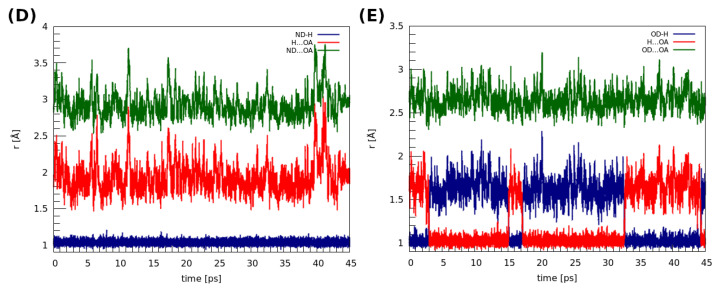
Time evolution of the metric parameters between the atoms involved in the hydrogen bond formation in the studied dimers. (**D**) 2,6-difluorobenzamide, (**E**) 4-hydroxybenzoic acid.

The structures of the dimers discussed in this section were extracted from the 919,101 and 908,103 deposits in the CCDC database [51]. CPMD, as well as PIMD calculations, were performed to shed light on the dynamic features of the intermolecular hydrogen bridges present in these structures (see Figure 2). The time evolution of the metric parameters of the hydrogen bonds is presented in Figure 5. Let us start the discussion with the dimer (D). In this case, the amide nitrogen is a proton donor, whereas the oxygen from the amide group serves as the proton acceptor. The distances between the donor and the acceptor of the hydrogen atom varied between ca. 2.6 and 3.9 Å, whereas the H…OA distance changes were within 1.5–3.0 Å. Throughout the whole simulation time, the hydrogen atom is located at the proton donor side. In the case of (E), where the oxygen atoms belonging to the carboxylic groups are the proton donors and acceptors, the observations that can be made are strikingly different. Here, the bridged proton freely switches its donor from one to another; proton transfer actually happened 3 ps after the production run of the dynamics started. Moreover, the proton was on both the donor and acceptor sides for a similar amount of time during the MD run. This substantial difference in the behaviors of the hydrogen bridges between these two dimers can be attributed to the differences between the amide and carboxylic groups, the electronegativity of the donor atoms as well as the impact of fluorine substituents in the structure of (D). We will come back to this issue later, when the electronic structure of both dimers will be studied in a more detailed way via the static approaches. In particular, the apparent large difference in strength of the hydrogen bonds in (D) and (E), leading to such different dynamic characteristics, will be confirmed by the QTAIM descriptors.

**Figure 6:**
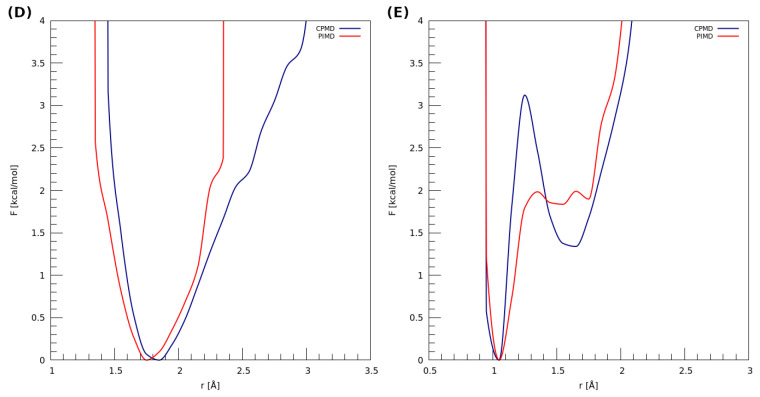
Potential of mean force (PMF) for the proton motion in the hydrogen bond of examined dimers with respect to the distance between the proton and its acceptor. (**D**) 2,6-difluorobenzamide and (**E**) 4-hydroxybenzoic acid.

The estimation of the impact of the nuclear quantum effects (NQEs) on the proton was possible based on PIMD—the obtained results are presented in Figure 6. It can be seen that, in the case of dimer (D), using the quantum-classical isomorphism to impose the quantization on the nuclei does not significantly change the proton behavior. The PMF profile corresponding to the PIMD simulation resembles the classical harmonic one. The observations made for the dimer (E) show that the NQEs lower the barrier for the proton transfer (from ca. 3.1 kcal * mol^−1^ to 2.0 kcal * mol^−1^) and predict two proton minima at the donor side, roughly at 1.55 Å and 1.70 Å of the H…OA distance. In the case of classical-quantum dynamics, we can observe two minima: one that occurs at ca. 1.05 Å, which is in accordance with the PIMD result, and the other one, which corresponds to 1.65 Å of the H…OA distance (proton at the donor side).

### 3.2. Electronic Structure Topological Analysis on the Basis of Quantum Theory of Atoms in Molecules (QTAIM)

**Figure 7:**
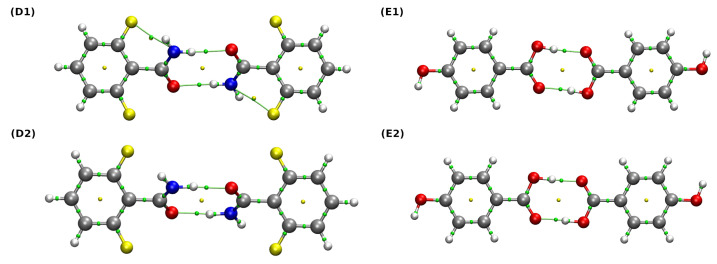
QTAIM molecular graphs of the studied dimers. Ball and stick model was used for visualization. Bond paths, BCPs and RCPs are presented as green lines and small green and yellow spheres, respectively. Color coding: white—hydrogen, grey—carbon, red—oxygen, blue—nitrogen, yellow—fluorine. (**D1**,**E1**) are the experimental structures, whereas (**D2**,**E2**) are the structures after the relaxation at the ωB97XD/def2-TZVP level of theory.

Quantum theory of atoms in molecules (QTAIM) served as a method of choice to investigate the electronic properties of the studied molecules. The visualization and the results concerning the covalent and non-covalent interactions of the experimental and optimized structures of the studied dimers are presented in Table 2 and in Figure 7.

The use of the QTAIM method allowed a qualitative and quantitative description of the interactions between monomers. Bond energies were estimated on the basis of their linear dependence on the potential energy density at the BCP (V_BCP_) via the Espinosa equation [95]. It can be observed that, in the case of both (D) and (E) dimers, the hydrogen bonds form quasi-rings, which provides structural stabilization. Their formation is indicated by the presence of the ring critical points (RCPs) (marked as small yellow spheres in Figure 7). For the dimer denoted as (D1), the properties at BCPs corresponding to two non-covalent interactions and two covalent bonds: ND-H…OA hydrogen bond and the intramolecular F…N contact as well as the ND-H and C=OA bonds were analyzed extensively. Both aforementioned non-covalent interactions can be considered weak, rather electrostatic in nature, since their estimated bond energies lie below 4 kcal * mol^−1^ and their Laplacian and energy density values are positive. Interestingly, when we inspect the data corresponding to the (D2) dimer, it can be seen that the relaxation of the structure causes the weakening of ND-H covalent bond, the intramolecular F…N interaction (which is not even detected by QTAIM) and the accompanying strengthening of the ND-H…OA hydrogen bond. In this case, the character of the ND-H…OA is noticeably more covalent, because the corresponding ρ and energy density values become larger and lower, respectively (H_BCP_ is indeed very close to zero) [34]. A similar analysis regarding the dimer (E) leads to the same conclusions: the optimization of the examined structures results in the strengthening of the hydrogen bond and the weakening of the corresponding covalent bond of the proton donor. More interesting is the characteristics of the hydrogen bond itself, namely OD-H…OA. In both the experimental and the optimized structures ((E1) and (E2)), the H_BCP_ values corresponding to the hydrogen bonds are negative and their bond energies are much larger compared to (D1) and (D2)—for this reason, one can say that they are more covalent in nature than their counterparts, ND-H…OA.

### 3.3. Decomposition of the Interaction Energy Using Symmetry-Adapted Perturbation Theory (SAPT)

Further insight into the nature of the interaction of the examined dimers was gained using the symmetry-adapted perturbation theory (SAPT) framework. SAPT is an invaluable method when it comes to the analysis of the interacting molecular fragments and their decomposition into physically grounded contributions. In the investigated dimers (see Figure 2), almost the whole interaction energy is constituted of two corresponding hydrogen bridges (results are presented in Table 3).

Let us start the discussion with the results obtained for the experimental structures of the analyzed dimers. Starting with (D1), it can be seen that the electrostatic term is almost counterbalanced by the exchange term—the sum of these contributions can be viewed as the electrostatic interaction of the symmetry-adapted reference state. Furthermore, for (D1), all of the contributions to the interaction energy are important; nonetheless, when we take into account their magnitudes, the dispersion turns out to be the most significant one. In the case of (E1), the sum of E_elst_ and E_exch_ is positive and thus repulsive. Due to this, the interaction energy between two monomers can be ascribed to the presence of the E_ind_ and E_disp_ terms.

The optimized structures ((D2) and (E2)) exhibit lower values of the total interaction energies. However, noteworthy is the fact that, for the optimized structures, the induction and the dispersion are, from a certain point of view, the most essential contributions to the interaction energies, respectively. It is evidently more visible for the (E2) structure and its corresponding HBs, which are more covalent in nature than their counterparts in (D2)—as such, the results obtained using the SAPT2+/aDZ level of theory are in accordance with the QTAIM method and agree with the electrostatic-covalent H-bond model proposed by Gilli [96]. For both experimental and optimized structures, the E_ind_ and E_disp_ terms play more significant roles for the dimer denoted as (E). Furthermore, both the Espinosa equation and the total energy obtained at the SAPT2+/aDZ level of the theory indicate that the OD-H…OA interaction strength is greater than its counterpart in (D), ND-H…OA. This difference is more pronounced in the results of the Espinosa equation, because it only covers the BCP of hydrogen bonding, while the SAPT method recognizes contributions from monomers, including the polarization of aromatic rings and other factors not directly related to hydrogen bonding.

## 4. Conclusions

The nature of intermolecular hydrogen bonds in exemplary compounds derived from benzamide, quinoline and benzoic acid groups was investigated. Quantum-chemical simulations were performed in vacuo and in the crystalline phase allowing a more in-depth analysis of the non-covalent interactions present in the chosen compounds for the current study: 2,6-difluorobenzamide, 5-hydroxyquinoline and 4-hydroxybenzoic acid. The computed power spectra of the atomic velocity reproduced the spectroscopic features of the investigated compounds indicating regions with O-H, N-H and C-H stretching. The comparison was made between the gas and crystalline phases, indicating the formation of the intermolecular hydrogen bonds. The metric parameter analysis based on the Car–Parrinello molecular dynamics results showed that the proton transfer phenomenon occurs for 4-hydroxybenzoic acid in the crystalline phase. Based on the results of the PIMD method, it was noted that the inclusion of quantum effects in the description of hydrogen bonds is important for strong interactions. Furthermore, theoretical investigations on the basis of static approaches (QTAIM and SAPT methods) revealed the strength of the non-covalent interactions as well as energy components. Hydrogen bonding energies estimated according to Espinosa’s formula indicate that, in the studied dimers, the interaction is stronger in 4-hydroxybenzoic acid. Finally, the SAPT results provided a detailed look inside the energy components of the intermolecular interactions. It was shown that the dispersion and the induction contributions to the interaction energy are decisive factors in the intermolecular hydrogen bonds studied herein. The HBs strength is strongly correlated with their covalency.

## Figures and Tables

**Table 1:** CCDC code and unit cell data for the investigated compounds.

Designation	CCDC Code	Unit Cell Data
(A)	919101 [51]	Monoclinic
		a = 5.139 Å, b = 12.118 Å, c = 11.792 Å
		β = 112.482${}^{\circ }$, Z = 4
(B)	908102 [51]	Orthorhombic
		a = 3.835 Å, b = 12.718 Å, c = 14.067 Å, Z = 4
(C)	908103 [51]	Triclinic
		a = 6.910 Å, b = 12.289 Å, c = 12.647 Å
		α = 112.713${}^{\circ }$, β = 93.424${}^{\circ }$, γ = 103.103${}^{\circ }$, Z = 2

**Table 2:** QTAIM-derived properties at BCPs for examined dimers from the structure deposited in the CCDC database and after geometry optimization at the ωB97XD/def2-TZVP level of theory. E1 is a bond energy based on the Espinosa model, given in kcal * mol^−1^. Units of gathered quantities are as follows: electron density, $\rho_{BCP}$, is given in $e\cdot a_{0}^{-3}$ atomic units and the Laplacian of electron density, $\nabla^{2}\rho_{BCP}$, is in $e\cdot a_{0}^{-5}$ units. V_BCP_ stands for BCP potential energy density and H_BCP_ denotes the energy density at the BCP.

System	BCP	ρ	V_BCP_	$\nabla^{2}$ ρ	H_BCP_	E1
		**Experimental Structure**				
**(D1)**	ND-H…OA	0.0183	−0.0124	0.0790	0.0037	3.9029
	F…N	0.0110	−0.0089	0.0601	0.0031	2.7829
	ND–H	0.4652	−1.0418	−3.5875	−0.9694	–
	C=OA	0.4046	−1.3240	−0.5213	−0.7272	–
**(E1)**	OD-H…OA	0.0384	−0.0381	0.1349	−0.0022	11.9557
	OD–H	0.4178	−0.9894	−3.3106	−0.9085	–
	C=OA	0.4069	−1.3567	−0.4383	−0.7331	–
		**Optimized Structure**				
**(D2)**	ND-H…OA	0.0302	−0.0254	0.1031	0.0002	7.9586
	ND–H	0.3229	−0.5649	−1.8740	−0.5167	–
	C=OA	0.4074	−1.3773	−0.4297	−0.7423	–
**(E2)**	OD-H…OA	0.0522	−0.0554	0.1237	−0.0122	17.3707
	OD–H	0.3092	−0.6476	−2.0757	−0.5833	–
	C=OA	0.4058	−1.3759	−0.4073	−0.7388	–

**Table 3:** Interaction energies between the examined dimers taken from the experimental structure [51] and after relaxation at the ωB97XD/def2TZVP level of theory. The calculations were performed at the SAPT2+/aug-cc-pVDZ level of theory, and the energies are given in kcal * mol^−1^.

Complex	E_elst_	E_exch_	E_ind_	E_disp_	SAPT2+/aDZ
		**Experimental Structure**			
**(D1)**	−15.730	13.619	−4.656	−5.380	−12.146
**(E1)**	−27.448	33.520	−12.021	−8.788	−14.736
		**Optimized Structures**			
**(D2)**	−24.472	27.813	−10.886	−8.037	−15.582
**(E2)**	−35.921	46.756	−21.032	−10.774	−20.970

## Data Availability

Not applicable.

## Abbreviations

The following abbreviations are used in this manuscript:

BCP	Bond Critical Point
BSSE	Basis Set Superposition Error
CPMD	Car–Parrinello Molecular Dynamics
ELF	Electron Localization Function
HB	Hydrogen Bond
IR	Infrared Spectroscopy
MEP	Molecular Electrostatic Potential
NQE	Nuclear Quantum Effect
PBCs	Periodic Boundary Conditions
PIMD	Path Integral Molecular Dynamics
RCP	Ring Critical Point
RDG	Reduced Density Gradient
QTAIM	Quantum Theory of Atoms in Molecules
SAPT	Symmetry-Adapted Perturbation Theory
